# Transforming Health Care Through a Learning Health System Approach in the Digital Era: Chronic Kidney Disease Management in China

**DOI:** 10.34133/hds.0102

**Published:** 2023-12-19

**Authors:** Guilan Kong, Jinwei Wang, Hongbo Lin, Beiyan Bao, Charles P. Friedman, Luxia Zhang

**Affiliations:** ^1^ National Institute of Health Data Science, Peking University, Beijing, China.; ^2^ Advanced Institute of Information Technology, Peking University, Hangzhou, Zhejiang, China.; ^3^ Renal Division, Department of Medicine, Peking University First Hospital, Beijing, China.; ^4^ Yinzhou District Center for Disease Control and Prevention, Ningbo, Zhejiang, China.; ^5^ Ningbo Urology and Nephrology Hospital, Ningbo, Zhejiang, China.; ^6^ Department of Learning Health Sciences, University of Michigan, Ann Arbor, MI, USA.

## Introduction

The last 30 years have witnessed the development of evidence-based medicine. It helps to achieve best practice by incorporating best available evidence into everyday practice. Conventionally, best evidence is generated through clinical studies such as randomized clinical trials (RCTs) and synthesized by systematic review and meta-analysis. Compared to evidence generation, fewer activities are taken to promote evidence uptake in practice. There is a long time lag between evidence and practice, and it may take up to 17 years [1]. To close the gap, the concept of learning health system (LHS) was proposed in a roundtable on evidence-based medicine by the Institute of Medicine (IOM) in 2006, which provides a way to leverage data to learn knowledge and to feed it back to the frontline practice in real time [2].

Continuous improvement of health care in an LHS is implemented by multiple data–knowledge–practice learning cycles run simultaneously [3]. A data–knowledge–practice learning cycle is composed of data collection, data analysis, and knowledge transformation, where what happens in routine practice is captured by data collection, knowledge is generated via data analysis, and both locally generated and other-sourced knowledge are applied to practice through knowledge implementation. By leveraging advanced information technology (IT), an LHS can integrate research and practice seamlessly, and thus can improve health care at lower cost. During the coronavirus disease 2019 (COVID-19) pandemic, the LHS approach was employed to improve not only patient outcome but also clinician well-being [4]. The development timeline of LHS has been examined by McGinnis et al. [5] in 2021, and it was highlighted that the next 50 years will see substantial progress toward LHS.

Nowadays, although LHS has gained a wide acceptance in high-income countries, the studies of LHS in low- and middle-income countries are limited. In China, digitization has fueled the rapid accumulation of health data, but the value of big data in health has not yet been fully realized. The LHS approach, which provides a data-driven framework to help transform big data to knowledge, and to improve health care by putting the knowledge to practice, cannot come at a better time. From this perspective, we aim to illustrate how the LHS approach helps to improve chronic kidney disease (CKD) management in China.

## Current Status of CKD Care in China

CKD has become a global public health problem. The number of deaths due to CKD has risen from the 27th place in 1990 to the 12th in 2017. The Global Burden of Diseases study estimated that CKD would be the 5th cause of mortality in 2040. Zhang et al. [6] reported that the prevalence of CKD in China was 10.8% based on a national survey study in 2012, but the awareness of CKD in China was approximately 10% in the population as a whole [7]. Moreover, there is huge heterogeneity in the progression of CKD [8].

The China Kidney Disease Network (CK-NET) was established in 2014 to improve CKD care in China. Since that time, CK-NET has set up a robust CKD surveillance system for systematic collection, analysis, and interpretation of health data from various resources to keep up with the changing patterns of and treatments for kidney disease [9]. An annual report on the spectrum and prevalence of CKD in China, based on the CK-NET data, has been published since 2017. In addition to this routine reporting, several data-driven prognosis prediction models have been developed using the CK-NET data [10].

In addition to the work of CK-NET, studies based on other data sources have been conducted in China [11,12]. Although some evidence of disease burden and some data-driven prediction models have been generated from those CKD-related studies, few results of these studies have been put into practice, and the CKD care in China still faces the challenges of high prevalence, low awareness, and limited efficiency [7].

It follows that the integration of discoveries from CKD-related studies into clinical practice, to facilitate the management of CKD patients, is an important issue needing to be addressed in China. LHS, as a systematic approach to leverage data to improve health care, is a possible way to meet these needs.

## Transforming CKD Management through LHS in China

Yinzhou, a district of Ningbo in Zhejiang province, China, which has a Regional Health Information Platform (YRHIP) established in 2009, was selected as the region for pilot LHS implementation. The YRHIP routinely captures patient information from local hospitals, primary care units, and other health institutions.

Beginning in 2018, a local CKD surveillance and management platform, CK-NET-Yinzhou, has been established by storing, managing, and analyzing all CKD patients’ data extracted from the YRHIP, with the aim to facilitate local CKD care by embedding clinical decision support tools into the CK-NET-Yinzhou [13].

**Fig. 1.**
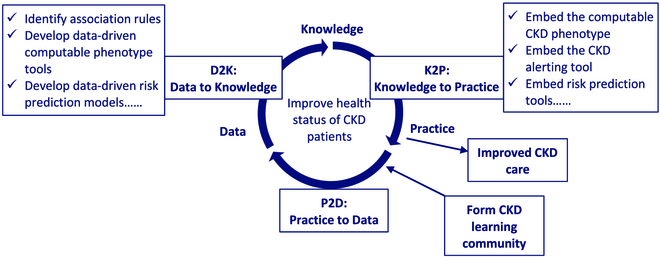
Data–knowledge–practice learning cycle in Yinzhou CKD LHS.

We sought to leverage these available data to improve the management of CKD in Yinzhou by implementing the LHS approach using a data–knowledge–practice learning cycle. How we did this is illustrated in Fig. 1.

The initial step in this process was to establish a CKD learning community composed of physicians from local hospitals, epidemiologists from local Center for Disease Control and Prevention (CDC), computer and data scientists from Peking University (PKU), and IT engineers from each local hospital information system. This community holds irregular meetings to learn the actual situation of CKD management in Yinzhou, and works together to identify CKD problems and to promote CKD LHS implementation in Yinzhou through embedding new software tools to the YRHIP.

The next task, as we entered the “practice to data” stage of the cycle, was the identification of CKD patients. Based on the electronic health record (EHR) data in YRHIP, a computable tool for identifying CKD patients was constructed and implemented by combining a common data model with a computable phenotype definition of CKD [14]. This process revealed that an estimated prevalence of CKD in Yinzhou was 12.0% [7], quite similar to that reported in the national survey. This also provided evidence of the validity of the computable phenotype used for CKD identification.

Based on this computable CKD identification tool, information about each CKD patient can be delivered in a timely fashion to the primary care unit serving that patient, enabling the physicians there to get in touch with these patients personally and advise them to seek medical service as soon as possible. This illustrates a rudimentary but important product of the data–knowledge–practice cycle whereby the CK-NET-Yinzhou data resource is converted to knowledge via the computable phenotype, which is then implemented by initiating appropriate care for individuals whose CKD may previously have been unknown or who, for other reasons, may not have been receiving appropriate CKD care.

In addition to facilitating early diagnosis and management of patients, the CK-NET-Yinzhou data resource also provides a platform for the validation, update, and implementation of some well-established risk prediction models for the incidence, prognosis, and various comorbidities of CKD. Once these models are validated for the local population, clinical decision support tools can be embedded in the YRHIP to help triage patients, prompt guideline-based suggestions, and link primary care with nephrology specialties, allowing for more efficient and effective management of CKD patients. Furthermore, comparative effectiveness studies could be taken to investigate the effectiveness of alternative treatments for CKD.

## Prospects for LHS Implementation in China

The regional CKD LHS, which was built on the basis of the CK-NET-Yinzhou, shows that the LHS approach is a feasible way to improve patient management by utilizing data-driven computable tools. It provides a good example for establishing LHSs in other geographic regions, in other health institutions, and for management of other diseases.

Building a successful LHS in health institutions faces challenges. In particular, a data-to-knowledge infrastructure, including EHR data and appropriately trained people, is required to generate evidence including rules, computable phenotypes, and risk prediction models. In the case of Yinzhou and CKD, such an infrastructure was already in place. Learning communities may be difficult to form if the potential members of the communities do not see value in the effort. Last, the evidence generated must be implemented. This will require changes in practice patterns, and it is universally known that professionals can be resistant to changing the way they do their work.

While high-quality RCTs are still the main mechanism generating clinical evidence, trials are expensive and time consuming, and the end of a trial is a journal article that may not, by itself, provide the means or motivation for practice change. Leaders of trials do hope that their evidence can be applied to practice, but strict patient inclusion criteria may have negative consequences on the real-world applicability and generalizability of the evidence generated by clinical trials. Also, the implementation of new evidence into practice is usually not part of a trial’s mission. By contrast, the LHS approach is built on a learning community committed to implementing what they discover. Leveraging its inherent relevance to routine clinical practice, LHS offers a dynamic and cost-effective approach to both generating and implementing evidence. This LHS pathway could be the key to accelerating the evidence to practice process, leading to better health for all people.

To summarize, although challenges exist, a well-constructed LHS infrastructure and the data–knowledge–practice learning cycle may advance evidence-based health care more rapidly and effectively than has previously been the case. In addition, digital health can be a transformational power for LHS establishment in practice by providing much of the data infrastructure that is needed for the LHS. With the aid of digital technologies, health data can be captured in real time, and patient health status can be assessed continuously. Combining digital health technologies with LHS is a promising way to transform health care in the near future.
